# Nighttime Foreground Pedestrian Detection Based on Three-Dimensional Voxel Surface Model

**DOI:** 10.3390/s17102354

**Published:** 2017-10-16

**Authors:** Jing Li, Fangbing Zhang, Lisong Wei, Tao Yang, Zhaoyang Lu

**Affiliations:** 1 School of Telecommunications Engineering, Xidian University, Xi’an 710071, China; fbzhang@stu.xidian.edu.cn (F.Z.); lswei@stu.xidian.edu.cn (L.W.); zhylu@xidian.edu.cn (Z.L.); 2 School of Computer Science, Northwestern Polytechnical University, Xi’an 710129, China

**Keywords:** nighttime foreground pedestrian detection, voxel surface model, near-infrared stereo network camera

## Abstract

Pedestrian detection is among the most frequently-used preprocessing tasks in many surveillance application fields, from low-level people counting to high-level scene understanding. Even though many approaches perform well in the daytime with sufficient illumination, pedestrian detection at night is still a critical and challenging problem for video surveillance systems. To respond to this need, in this paper, we provide an affordable solution with a near-infrared stereo network camera, as well as a novel three-dimensional foreground pedestrian detection model. Specifically, instead of using an expensive thermal camera, we build a near-infrared stereo vision system with two calibrated network cameras and near-infrared lamps. The core of the system is a novel voxel surface model, which is able to estimate the dynamic changes of three-dimensional geometric information of the surveillance scene and to segment and locate foreground pedestrians in real time. A free update policy for unknown points is designed for model updating, and the extracted shadow of the pedestrian is adopted to remove foreground false alarms. To evaluate the performance of the proposed model, the system is deployed in several nighttime surveillance scenes. Experimental results demonstrate that our method is capable of nighttime pedestrian segmentation and detection in real time under heavy occlusion. In addition, the qualitative and quantitative comparison results show that our work outperforms classical background subtraction approaches and a recent RGB-D method, as well as achieving comparable performance with the state-of-the-art deep learning pedestrian detection method even with a much lower hardware cost.

## 1. Introduction

Pedestrian detection is an important topic and can be applied to a variety of computer vision applications [1], such as intelligent video surveillance [2], autonomous service robots, human–computer interaction, self-driving cars and ADAS (Advanced Driver Assistant System) [3]. In general, detecting foreground pedestrians in the scene is a fundamental task in computer vision. First, pedestrian detection can significantly increase the understanding of the scene and directly extract the most important information for users. Second, most computer vision algorithms, from low-level image analysis to high-level recognition, usually depend on the detection result. Therefore, from the beginning of the development of computer vision to the present, there have been many pedestrian detection algorithms that have been proposed and with continuous improvement. However, most of these studies are carried out during the day with good illumination, and many daytime pedestrian detection systems cannot be used directly to detect pedestrians at night [4]. This is because much of the monitoring equipment has difficulty obtaining a clear on-site situation owing to the lack of a visible natural light source and the poor visibility of the camera at night. It is clear that pedestrian detection at night is essential; compared with the daytime, the light at night is weak, and nighttime is more prone to security incidents [5]. In fact, not only do treasuries, cultural relics departments and other important departments use night monitoring systems, but residential TV monitoring projects have also gradually added a night monitoring function, and there is a growing need for 24-h continuous monitoring at some important sites. Therefore, the pedestrian detection system at night is particularly important for the field of surveillance [6]. In the past several decades, the pedestrian detection method at night has garnered enthusiastic attention and interest [7]. Generally speaking, in the nighttime pedestrian detection system, researchers tend to employ an Infrared (IR) sensor to compensate for this lack of ambient illumination and to detect the IR heat signature generated by the human body [8]. Taking into account the means of image acquisition, the latest nighttime pedestrian detection systems can be divided into two categories.

One is passive, i.e., long-wavelength infrared (LWIR) systems: a far infrared imaging system works by sensing the temperature of an object. Recently, effective pedestrian detection algorithms using FIR cameras have been proposed. For example, Olmeda et al. [9] propose a new contrast-invariant descriptor for pedestrian detection in FIR images, which could detect pedestrians in images taken with an inexpensive temperature-sensitive uncooled microbolometer. Bavarian Motor Work (BMW) introduced a system called “NightVision” to the cars of the 7 series; this is a passive system [10]. The detection range is large, which is one of major advantages of passive systems. However, there are some disadvantages when using LWIR thermal sensors. For example, LWIR is low resolution and high cost; the LWIR image contrast varies depending on the ambient temperature, like in summer time, when the temperature of the environment is almost equal to the body temperature, which does not allow a pedestrian to be differentiated from the background [11].

Another kind of system is active, i.e., Near-Infrared (NIR): NIR pedestrian detection systems are able to detect a human because human skin reflects strong NIR light [12]. There are some effective pedestrian detection algorithms using NIR cameras, such as Lee [13], who proposes an NIR-based nighttime pedestrian detection system using the grouped part models method. This method aims to deal with the problem that the pedestrian is partially invisible because clothes on the pedestrian might absorb most of the infrared. Dong [14] presents a novel nighttime pedestrian detection approach with a near-infrared camera using cascaded classifiers, which can be used in practical driver assistance systems. The main advantage of active systems is the high resolution, and the image is easy to interpret for users due to the proximity of NIR to visible light. What is more, the NIR camera has a relatively low cost and small size, and it can also be used during the day (without NIR illumination) and at night (with NIR illumination), which makes it attractive and widely available.

**Figure 1:**
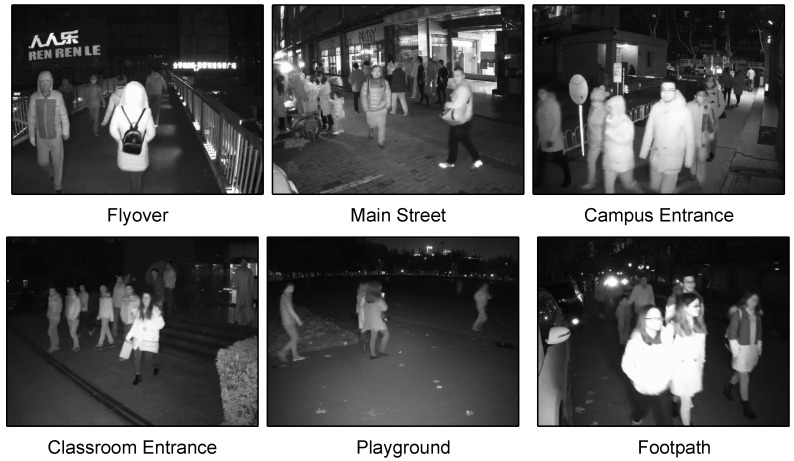
Examples of our stereo nighttime pedestrian dataset, which includes flyover, main street, campus entrance, building entrance, playground and footpath. Note that besides low illumination, less texture information and blurred contours, these scenes also contain severe mutual occlusion between the human bodies.

In other words, with the rapid progress of NIR camera performance, recently, more and more researchers have been inclined to select a near-infrared camera as the image acquisition equipment in a pedestrian detection system, especially in the field of monitoring. As shown in Figure 1, there are some common monitoring scenes including flyover, main street, campus entrance, building entrance, playground and footpath. From these images, it can be seen that there are plenty of challenges to achieving an effective nighttime pedestrian detection system. Firstly, the color discrimination of the image is decreased because the illumination at night is weak [13]. Furthermore, owing to the gray value of the obtained image being dim, the image has less texture information and blurred contours. So it is difficult to distinguish between background and foreground targets or the foreground targets and other foreground targets at night. Secondly, there are serious occlusion problems in many crowded scenes, which cause parts of the pedestrians to be invisible in these scenes. Thirdly, most of the applications have several special requirements such as real time, low price and high detection accuracy. In the past few decades, a variety of methods has been presented in the literature to address those challenging issues. Below, three of the most popular methods as of now [15] are described.

Background subtraction-based detector: This method extracts the foreground moving object and determines whether it is a pedestrian, using the Gaussian Mixture Model (GMM) [16], the Visual Background extractor (ViBe) [17], fast MCD [18], etc. Although this method has the advantages of low computational complexity, simple implementation, fast calculation speed and is widely used in most cases, the technique must be able to adapt to the changes of ambient brightness, camera motion and complex background objects in the scene. Furthermore, in the monitoring of outdoor scenes, the robustness against noise and the adaptability to light changes is also necessary. In short, this method has satisfactory results when the background is relatively simple, but its performance often deteriorates when the background becomes complex.

Learning-based detector: This is a popular method of pedestrian detection, according to a large number of samples for building a pedestrian detection classifier. The extracted features are gray level, edge, color, gradient histogram and other information [19]. The classifier mainly includes the Histogram of Oriented Gradient (HOG) detectors [20], the Deformable Parts Model (DPM) [21] detector [21], the deep learning human detector [22,23,24], such as the Single Shot multibox Detector (SSD) [25], You Only Look Once Version 2 (YOLO2) [26], faster Regions with Convolutional Neural Networks (RCNN) [27] and the multispectral human detector [28]. Although such algorithms have achieved marvellous success when the human appearance has model consistency and complies with the design of the detector, there still exist two main problems that have to be solved when implemented on pedestrian detection tasks. Firstly, the performance of this classifier is greatly affected by training samples. Secondly, the performance can be significantly reduced in a scene with severe occlusion.

RGB-D-based Detector: The method of pedestrian detection directly from the original image cannot effectively solve occlusion in some environments. To overcome this limitation, researchers notice that the depth information is beneficial to deal with occlusion. The RGB-D method is always used with other methods and divided into two categories according to the method of obtaining the depth map. One is RGB-D-based pedestrian detection [29,30]. This method obtains object depth with an RGB-D sensor, such as Kinect [31]. However, this method generally cannot be used for outdoor pedestrian detection because the camera is sensitive to light. The other one is stereo pedestrian detection [32]. This method is more flexible and can be used in outdoor environments due to passive imaging. Now, it is convenient to obtain the disparity map of a scene because there are many stereo matching algorithms, such as Boyer–Moore (BM) [33], Semi-Global Matching (SGBM) [34] and Efficient Large-Scale Stereo (ELAS) [35]. Therefore, this pedestrian detection method based on RGB-D is becoming more and more popular.

From previous studies, we know that a proper camera and an effective pedestrian detection algorithm are very crucial for a pedestrian detection system at night. In this paper, we present a novel nighttime pedestrian detection system based on the voxel surface model to detect pedestrians at night, which can solve the problem of partial occlusion and be used in a real-time system. The preliminary work of our pedestrian detection approach is firstly introduced in [15]. In this paper, we significantly improved pedestrian detection system, and the main contributions of this paper are as follows:

- First, in the system, we assemble a binocular camera to acquire stereo images at night. The characteristics of this camera mainly include two parts: (1) Our binocular camera is based on the network transmission, and it is convenient to be used in the far distance surveillance scenarios; (2) The binocular camera is built with two less expensive Near-Infrared cameras and LED lights, thus it can generate the stereo images in real nighttime monitoring scenes. The parameters of our binocular camera are shown in Table 1.
- Second, we showcase a voxel surface model to detect pedestrians at night. This method is based on the three-dimensional spatial structure information and needs a new background surface model updating strategy, which is a free update policy for unknown points, which is essentially different from the traditional background subtraction method based on the gray value space.
- Third, we built a new stereo night dataset; to the best of our knowledge, this is the first night stereo video surveillance dataset to address and analyze the performance of state-of-the-art pedestrian detection algorithms. There is a large number of experimental results that show that this system can solve the problem of partial occlusion and is not very sensitive to light intensity in night scenes. What is more, this method is fast and can meet the real-time requirements of our monitoring system and our output results are accurate. In addition to the detection of the bounding box, the foreground binary image and foreground cluster depth are also detected.

## 2. Voxel Surface Model for Foreground Pedestrian Detection

**Figure 2:**
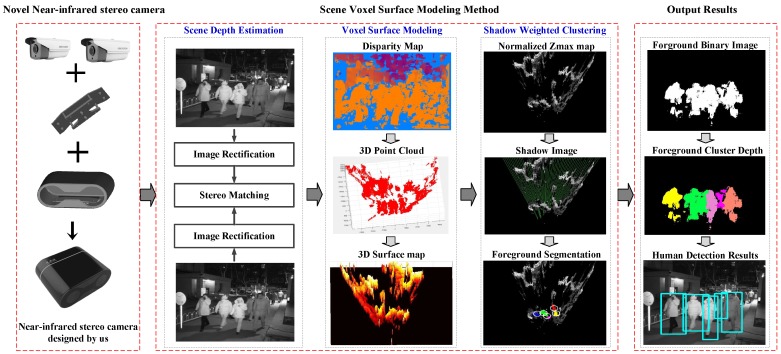
The framework of the proposed nighttime pedestrian detection algorithm and system. (**Left**): We build a network binocular camera with two calibrated commercial near-infrared cameras; (**Middle**): The voxel surface model for foreground pedestrian detection in dynamic scenes; (**Right**): Three kinds of outputs of our system, including the foreground binary image, foreground cluster depth and bounding boxes of the pedestrian.

In this section, we develop a robust pedestrian detection system at night based on a novel voxel surface model. When we design the entire system, in order to promote this system for business applications in the future, we take into account a number of factors such as the price of the camera, the configuration of the camera, the overall system structure, the robustness of the algorithm, etc. An overview of the proposed method is illustrated in Figure 2.

Our system mainly includes three parts. First, we choose a self-built binocular stereo NIR camera to acquire images, which is composed of the two ordinary cameras with the near-infrared function. The price is much less than thermal infrared cameras (as given in the left module of Figure 2). Second, we propose a novel voxel surface model method to detect pedestrians at night. What is more, we introduce a new background surface model-updating strategy, which is a free update policy for unknown points, and in foreground segmentation, we use shadow as the target’s prior condition to decrease the false alarm rate. The algorithm flow is shown in the middle module of Figure 2. Finally, the output results are shown in the right module of Figure 2. We know this system can obtain a variety of output results, including foreground binary image, foreground cluster depth and human detection results, and the results of the detection confirm that our system can be applied to actual life and has a robust detection performance.

### 2.1. The Binocular NIR Camera

In this part, we mainly talk about a new binocular NIR camera, as shown in Figure 2. One of the basic steps of the system is to construct the optical imaging equipment. Good optical imaging equipment will make the object in the image prominent, simplify the algorithm, guarantee the stability of the system, etc. In this system, we assemble a new binocular camera to acquire images, which is built using two less expensive network near-infrared cameras, and the parameters of this camera are given in Table 1.

At present, one of the popular binocular cameras on the market is the BumbleBee XB2, having a firewire to get the images. The other one is ZED stereo camera, having a USB interface to connect the computer. The transmission distance of these cameras is limited, and they generally work in the daytime owing to their visible imaging system. Therefore, they are not suitable for use in the field of video surveillance at night. There are some thermal infrared cameras on the market, but they are expensive and cannot obtain stereo images. Therefore, we build a binocular camera using two less expensive network NIR cameras, which can achieve long distance image transmission and obtain stereo images at night. The camera is an off-the-shelf product and is suitable for large-scale use of monitoring areas. From Table 1, the baseline distance of this camera is 190 mm, and the effective monitoring distance is about 15 m, noting that the baseline distance is very flexible and can be adjusted according to the actual monitoring distance. What is more, the binocular camera has the Infrared Cutfilter Removal (ICR) infrared filter. The filter works in the daytime, and the full spectrum optical glass works at night. Therefore, the camera could be used during the night monitoring after a natural transition, and it uses the CMOS image sensors, which have good image performance, high integration, low power consumption and fast speed. Furthermore, the camera connects to the computer through a network cable, so it is easy to acquire images through a small router, and it is convenient for use in many application scenarios.

### 2.2. Voxel Surface Model Generation

From the middle module of Figure 2, the vital step of this pedestrian detection method is the generation of the voxel surface map in the scene. In order to get the voxel surface model, we should firstly estimate the scene depth; see the left part in the middle module of Figure 2. First, we obtain the original images from the binocular stereo NIR camera; it should be noted that the original obtained images are gray images. Then, we use Zhang’s calibration method to get the parameters of this binocular camera, which are used to correct the original left and right images. Thereafter, we chose a stereo matching algorithm to compute the disparity map, and we evaluate many algorithms, such as ELAS [35], SGBM [34], BM [33], etc. The disparity maps of ELAS and SGBM are better and relatively dense, but their computing speed is slow in the CPU development environment. Because our goal is to design a real-time and robust pedestrian detection system, we prefer a simple and fast algorithm in the stereo matching module. After carefully designing the whole system, our algorithm does not have a strict requirement for the quality of the disparity map. We could achieve satisfactory performance even with the simplest BM, although BM is prone to producing many invalid points, and the processing speed of BM is over 80 fps, which is fast enough to meet the real-time requirements of our system. Therefore, the simplest and high-speed BM is chosen in this work to obtain the disparity map and the 3D coordinates in the camera coordinate system.

After we obtain the disparity map, we should calculate the coordinates of the 3D points and convert these points into a voxel surface map; see the middle part in the middle module of Figure 2. We manually adjust the external parameters of the camera and find the best projection surface to project the 3D coordinates in the camera coordinate system into the world coordinate system, and the requirements for these external parameters are not strict in our system. In this way, we can get the 3D coordinates (u,v,w) of all of the points in the world coordinates (see the 3D point cloud in Figure 2). Notice that these 3D points may be correct or invalid 3D, while no points are generated in some areas due to occlusion. Thereafter, for the convenience of computer processing, we quantify the 3D point cloud as a fixed-size discrete 3D space, and we assume that the size of this discrete space is W×H×Z. In this discrete space, every discrete point is treated as a small voxel (a volume of element, and the dv=dx×dy×dz). It is clear that there are many 3D points (u,v,w) in the world coordinates that will be quantified as one discrete point (x,y,z), and it can be said that they belong to the same voxel model in the discrete 3D space; such that one person should be composed of many such voxels in a fixed-size discrete 3D space. In this system, we define every pixel voxel model by (x,y,z), which means the point p(x,y,z) in the discrete 3D space. (x,y) is the voxel position coordinates, and x∈[0,W−1],y∈[0,H−1]; the z value presents the height of this voxel position and we quantify the *z* value to [0,255]. Remember that invalid 3D points and occluded regions are unknown in 3D space, and we think their pixel voxel model is (x,y,0) (see the 3D surface map in Figure 2).

If we estimate every voxel model in 3D space, we can imagine that it is a very heavy burden in time consumption. What is more, we take into account that people are higher than the ground in the actual situation and that human movement always changes the position of the highest point in the scene. Therefore, in this paper, we pay more attention to the surface of the voxel model depending on the height information. In every location of (x,y), we just estimate the highest voxel model, then we can get the voxel surface map, which is our voxel surface model. In this system, we use a gray image named the Zmax map to describe the voxel surface model, and we define the size of the Zmax map as W×H, and the gray-scale value of point (x,y) is Zmax(x,y) (see the Zmax map in Figure 2). It is obvious that the gray value Zmax(x,y) in the Zmax map represents the height of the highest voxel model in position (x,y) in the 3D discrete space. If we assume that $U=p\left(x^{\prime },y^{\prime },z^{\prime }\right),x^{\prime }\in \left[0,W-1\right],y^{\prime }\in \left[0,H-1\right],z^{\prime }\in \left[0,255\right]$ is a collection of all voxel models in the 3D discrete space and there are many different height voxel in the location of (x,y), the height of the highest voxel Zmax(x,y) is:

(1) $$Zmax\left(x,y\right)=\{max\left(z^{\prime }\right)|\left(p\left(x,y,z^{\prime }\right)\in U,x\in \left[0,W-1\right],y\in \left[0,H-1\right]\right\}$$

### 2.3. Voxel Surface Background Model Establishment and Updating

After obtaining the voxel surface model of the scene, that is the normalized Zmax map (as shown in Figure 2), when there are moving objects in the monitored scene, the highest point of the scene will change, and the voxel surface model will change. In general, the place where the model changes is the position of the moving object, and this means the place where the gray value changes is the position of the moving object on the Zmax map. In our system, we use this voxel surface model to extract and locate foreground moving objects in the scene, so we first consider the establishment of the voxel surface background model, and this model should be adaptive to gradual or fast illumination changes and motion changes. For example, GMM [16] uses a weighted mixture of Gaussian functions to characterize the features of each background pixel whose distribution is observed over time. ViBe [17] models each background pixel with a set of samples instead of with an explicit pixel model, etc. In the experiment, we chose to build our random sampling-based voxel surface background model by imitating ViBe [17].

Formally, we know Zmax(x,y) is a given gray value taken by the point located at (x,y) on the Zmax map at time *t*, and let us denote by $Zmax_{\left(i\right)}$ a background sample value randomly selected with an index *i*. In the system, we select samples randomly in the eight-connected neighborhood of each point, so each background point (x,y) is modeled by a collection *N* of background sample values taken in the previous frames, and the voxel surface background model M(x,y) of each point is:

(2) $$M\left(x,y\right)=\left\{Zmax_{\left(1\right)},Zmax_{\left(2\right)},\ldots ,Zmax_{\left(N\right)}\right\}$$

Note that it is possible for a given $Zmax_{\left(i\right)}$ to be selected several times (for example, if the size of neighborhood is smaller than N) or to not be selected at all. Then, we introduce the voxel surface background model initialization. Many popular techniques need a sequence of several dozens of frames to initialize their models, and in this experiment, the background model is initialized by using the single frame video sequence, which is consistent with ViBe [17]. Formally, we assume that t=0 indexes the first frame and that NG(x,y) is a spatial neighborhood of a point location (x,y). Therefore,

(3) $$\begin{array}{cc} M^{0}\left(x,y\right) & =\{Zmax(x,y)|(x,y)\in NG(x,y)\}\\ & =\{Zmax_{\left(1\right)}^{0},Zmax_{\left(2\right)}^{0},\ldots ,Zmax_{\left(N\right)}^{0}\} \end{array}$$

where locations are chosen randomly according to a uniform law.

After that, we describe how to continuously update the voxel surface background model with a new frame. The update process must be able to adapt to various light changes at night and to handle new objects that appear in a scene. The classical method of updating the background is to replace the old background value with a new background value after multiple frames or after a given period of time (usually about a few seconds) [17]. In our method, we can get the voxel surface background model set $M^{t}\left(x,y\right)$ of point (x,y) at time *t*, and our update strategy is similar to ViBe [17] as follows.

(1) Conservative update policy: Foreground points will never be used to update the voxel surface background model.
(2) Time subsampling: For a point (x,y) that is classified as background in time *t*, it has a probability of 1/ϕ to update its own model $M^{t}\left(x,y\right)$, and ϕ is a time subsampling factor.
(3) Spatial consistency through background sample propagation: the point (x,y) has a probability of 1/ϕ to update its neighboring points’ background model.
(4) Memoryless update policy: Every time the voxel surface background model $M^{t}\left(x,y\right)$ is updated, the new point value Zmax(x,y) will replace one sample randomly chosen from $M^{t-1}\left(x,y\right)$.

In addition, a new updating strategy, which is a free update policy for unknown points, is introduced in our algorithm. Unknown points occur due to invalid points or areas where no points were generated due to occlusions. It is clear that the gray value of these unknown points on the Zmax map is zero. The Zmax map is shown in Figure 2. We can find that there are many unknown points (x,y) whose gray value Zmax(x,y) is zero, and these points can be divided into two cases. First, we can see there are a large number of regions behind the high targets, and these regions are not observable due to occlusion. Second, we can see some scattered invalid points where the disparity values are computed wrongly. In other words, we do not know the 3D information of these unknown points, and we are unaware of whether the background changes in these points. Therefore, these unknown points cannot be used to update the background model. More formally, in every position of (x,y) on the Zmax map, we assume that the background model of (x,y) at time t−1 is $M^{t-1}\left(x,y\right)$, for a point (x,y) that is classified as a background point in time *t*. Then, $M^{t}\left(x,y\right)$ is:

(4) $$\left\{\begin{array}{c} {\dot{M}}^{t-1}\left(x,y\right),if\;Zmax\left(x,y\right)=0\\ update\;M^{t-1}\left(x,y\right),if\;Zmax\left(x,y\right)\neq 0 \end{array}\right.$$

### 2.4. Shadow Extraction

In this part, we extract the shadow of foreground objects on the Zmax map [15]. The shadow image is as shown in the middle module of Figure 2. Along the camera’s viewing degrees, there are invisible areas occluded by foreground objects, and we call these areas the shadow of these objects in the scene. Therefore, it is easy to know that the shadow area exists in the scene no matter whether day and night, and the length and shape of the shadow area are usually different. In the system, the shadow is composed of some areas where no points were generated due to occlusions, and the gray value of the shadow on the Zmax map is zero. However, we know there is a large region for which the gray value Zmax(x,y) is zero in this map (see the Zmax map in Figure 2), and there are two cases of these areas. One case is that these areas are composed of some invalid points, and these areas are usually very small. The other case is that these areas are composed of some points that cannot observed; these areas are actually shadows of foreground objects, so we mainly discuss the second case.

**Figure 3:**
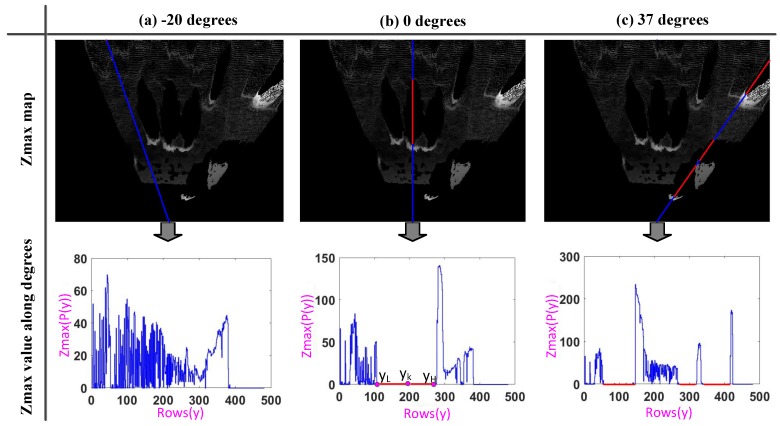
We select the three angles to illustrate the way of finding the shadow on the Zmax map. The blue line represents the route of looking for shadows at a camera’s viewing degrees, which is a ray. The red line indicates the shadow found at this angle. What is more, we could get the change curve of the Zmax value along this degree, and the three features of the shadow are clearly shown.

Now, we introduce how the shadow area is extracted on the Zmax map. As shown in Figure 3, we select three angles, such as −20${}^{\circ }$, 0${}^{\circ }$ and 37${}^{\circ }$, to illustrate the way of finding the shadow on the Zmax map. On the Zmax map, the camera’s viewing direction is a ray that is emitted along the point $\left(\frac{W}{2},H-1\right)$; in the system, *W* and *H* are 500. We define R(Ang) as representing the set of all points on the ray whose angle is Ang, and row by row to search for points P(x,y) on this ray. More specifically, we know when y=H−1,H−2,…,0, x=tan(Ang)×(H−1−y)+W/2, because x∈[0,W−1],y∈[0,H−1], so we define $\left(x^{\prime },y^{\prime }\right)$ as the end of the ray, and $y^{\prime }$ is:

(5) $$\left\{\begin{array}{rr} \overset{}{t},if\;\exists t\in & \left[0,H-1\right],when\;y\in \left[0,t\right),x\notin \left[0,W-1\right]\\ & 0\;,if\;when\;y\in [0,H-1],x\in [0,W-1] \end{array}\right.$$

Therefore, the ray is represented as:

(6) $$R\left(Ang\right)=\{P\left(x,y\right)|y\in \left[y^{\prime },H-1\right],x=tan\left(Ang\right)\times \left(H-1-y\right)\times 2+W\}$$

or simply $R\left(Ang\right)=\{P\left(y\right)|y\in \left[y^{\prime },H-1\right]\}$. Then, we define S(Ang) as representing a collection of shadow area points at this degree, and it can be written as:

(7) $$S\left(Ang\right)=\{P\left(y_{1}\right),P\left(y_{2}\right),\ldots ,P\left(y_{k}\right)|P\left(y_{k}\right)\in R\left(Ang\right)\}$$

At this time, we could get the change curve of the Zmax value along degree Ang in Figure 3, and from the changes of Zmax(P(y)), we know that there are three important characteristics of the shadow area:

(1) The point $P(y_{k})$ where the gray value is zero on the Zmax map, which means $Zmax(P\left(y_{k}\right))=0$.
(2) The front of the shadow area must have a relatively high target. This means that there are some points with a larger gray value in front of the shadow area on the Zmax map, so the point $P(y_{k})$ must satisfy the following condition: ∃P(y)∈R(Ang), making Zmax(P(y))>0 and $Zmax\left(P\left(m\right)\right)=0,y_{k}\leq m<y$ and marking y−1 as $y_{H}$, as (b) in Figure 3.
(3) The shadow area cannot be too small. We know there must ∃P(y)∈R(Ang), making Zmax(P(y))≥0 and $Zmax\left(P\left(n\right)\right)=0,\;y<n\leq y_{k}$, and marking *y* as $y_{L}$, like (b) in Figure 3. Then, we can set a threshold Th to determine whether the size of the shadow area is acceptable. That is to say, the $P(y_{k})$ must satisfy the following condition: $y_{H}-y_{L}>Th$.

**Figure 4:**
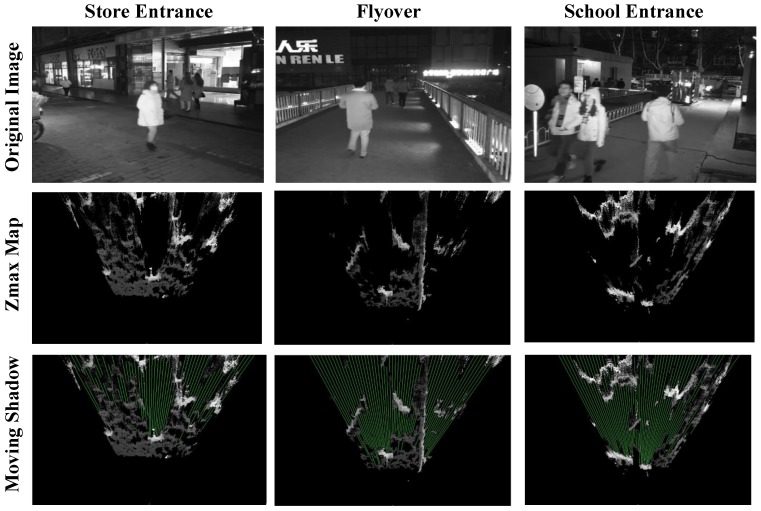
Examples of moving shadow segmentation under various outdoor scenes at night. Please note that by detecting motion shadows, our system can use the shadows to remove some false targets by judging whether there is any shadow behind the detected foreground pedestrian and more accurately detect targets in dim lighting of various occlusion scenes.

Therefore, we can find the shadow area along the camera’s viewing direction according to these three characteristics (as given in Figure 4), and the shadow is used in the foreground pedestrian segmentation.

### 2.5. Foreground Extraction and Pedestrian Segmentation

In this section, we explain how to detect the foreground points in a new frame. We could see the problem of background subtraction as a classification problem, so in order to classify a new point (x,y) in a new frame at time *t*, we compare how it fits within the estimated voxel surface background model. The point (x,y) is classified as background including three cases:

(1) The first one is Zmax(x,y)=0, which means point (x,y) belongs to the unknown region.
(2) The second is when:
  (8) $$Zmax\left(x,y\right)<Ra(M^{t-1}\left(x,y\right))$$
  $Ra(M^{t-1}\left(x,y\right))$ represents one background point sample value that is randomly extracted from the voxel surface background model at location (x,y), so $Ra\left(M^{t-1}\left(x,y\right)\right)\in M^{t-1}\left(x,y\right)$. We think that $Ra(M^{t-1}\left(x,y\right))$ could represent the height of its corresponding voxel surface background model at time (t−1). Therefore, this situation means the height of the new point (x,y) below the voxel surface background model at the location (x,y).
(3) The final case is a point (x,y) belonging to the background model $M^{t-1}\left(x,y\right)$ if the following condition is satisfied:
  (9) $$\begin{array}{c} ♯\{S_{R}\left(Zmax\left(x,y\right)\right)\cap \left\{Zmax_{\left(1\right)}^{t-1},Zmax_{\left(2\right)}^{t-1},\ldots ,Zmax_{\left(N\right)}^{t-1}\right\}\}\geq C \end{array}$$
  $S_{R}\left(Zmax\left(x,y\right)\right)$ denotes a sphere region centered at Zmax(x,y) with a radius *R*; the operator ♯ denotes the cardinality of a set; and *C* is a predefined constant and is used as a threshold for comparison. This formula involves the computation of *N* distances between Zmax(x,y) and model samples and of *N* comparisons with a thresholded Euclidean distance *R*. This formula indicates that the cardinality of the set intersection of this sphere and the background model sample set is larger than or equal to a given threshold *C*.

To be more specific, the first case is because we think the foreground objects can always be observed, so the unknown points should be classified as background. In the second case, we classify this point as background because the highest point of the foreground target is always higher than the highest point of the background model. Therefore, this foreground extraction strategy could be used to avoid generating ghosts when a person who is updated to the background moves suddenly. The final case means the height of this point is similar to the height of its corresponding voxel surface background model, so this point is background. In this way, we could get all of the foreground points (x,y) on the Zmax map, and we also get all of the foreground points (x,y,z) in the voxel model. Then, we project these 3D points back onto the two-dimensional image to acquire the foreground binary image in the scene.
**Algorithm 1:** voxel surface modeling
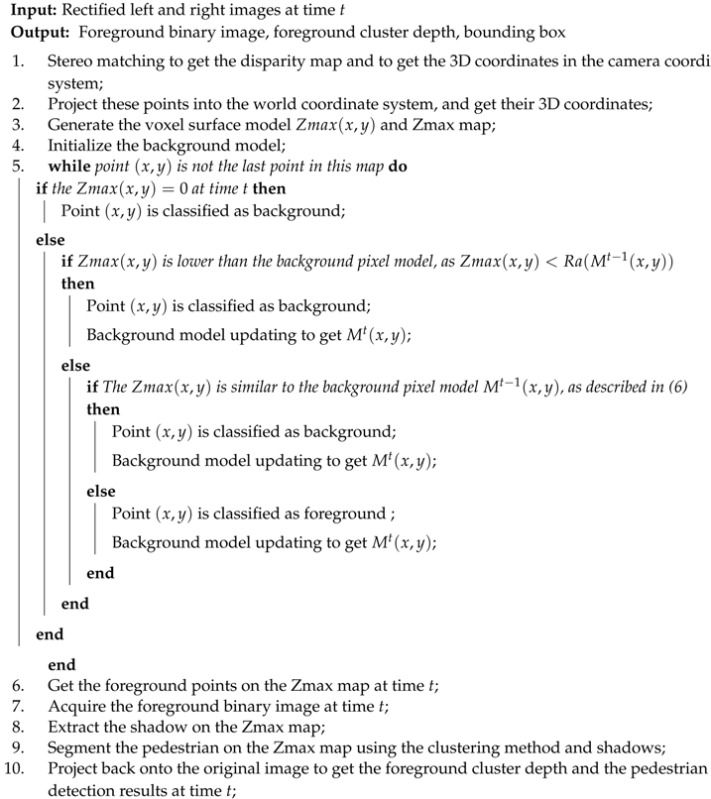


After that, we can segment the foreground points to locate foreground objects. Although the disparity map at night due to dim light will have many invalid points, most points of the moving target will be detected by the near-infrared camera. From the Zmax map, we can see that each foreground pedestrian has a certain amount of points gathered together in this image, and the gray value of these points is higher than the surrounding points on the Zmax map. Therefore, we choose a simple clustering method to segment the foreground pedestrians; in this system, we select mean shift [36] as the clustering method. In the clustering process, first of all, we choose the cluster on the Zmax map and the extracted foreground points as clustering centers so as to more easily cluster the targets and separate the two partially overlapping targets. Then, we select the appropriate clustering window width *w* according to the size of the pedestrian. In this way, we can quickly locate all of the foreground pedestrians. More formally, we know point (x,y) is a foreground point, so we choose point (x,y) as the clustering center and set the clustering window width to *w*. The iterative formulas are:

(10) $$\begin{array}{c} x^{\prime }=\frac{\sum_{(x,y)\in w}x\cdot Zmax{\left(x,y\right)}^{p}}{\sum_{(x,y)\in w}Zmax{\left(x,y\right)}^{p}} \end{array}$$

(11) $$\begin{array}{c} y^{\prime }=\frac{\sum_{(x,y)\in w}y\cdot Zmax{\left(x,y\right)}^{p}}{\sum_{(x,y)\in w}Zmax{\left(x,y\right)}^{p}} \end{array}$$

We can change the weight of the cluster by adjusting the *p*-value. In our algorithm, we set *p* as two. The foreground segmentation of this system is shown in the middle module of Figure 2, and from the clustering results, it can be seen that there are five moving targets detected. After the foreground segmentation, there are many noises in the image, and these noise points will also be classified as foreground. At this time, the mentioned extracted shadow can be utilized to remove noise points and keep the targets by judging whether there is any shadow behind the detected foreground pedestrian. Based on this observation, we can significantly reduce the false alarms. In this way, we can locate the pedestrian on the Zmax map. Thereafter, we project back onto the original image to get the pedestrian detection results, the foreground binary image and the foreground cluster depth, and all output results are as shown in the right module of Figure 2. We summarize the voxel surface modeling in Algorithm 1.

## 3. Experimental Results

### 3.1. System Setup and Nighttime Stereo Dataset

In this section, in order to evaluate the effectiveness and efficiency of the proposed nighttime pedestrian detection system, we build a complete nighttime pedestrian detection system that includes hardware and algorithmic parts. The hardware equipment included a binocular camera, computers, a network cable and brackets. The binocular camera was built using two inexpensive network cameras with the function of near-infrared by us, which served to acquire images in the scene, and our camera used a 190-mm baseline length, so the detected distance of this constructed pedestrian detection system was about 1–10 m. The computer was used to implement the algorithm to process the image from our binocular camera, and the computer connected with the camera through a network cable. As for the brackets, we fixed the camera on a bracket, so that we could easily move this device in different monitoring scenarios for data acquisition and system performance testing.

In the experiment, we tested our system and compared it with other methods for pedestrian detection in some challenging environments at night. At present, there are many public databases that can be used to test the pedestrian detection algorithms, but these databases are generally from monocular cameras during the day. Therefore, there are several binocular databases such as KITTI, but this database is used for a mobile platform, so our system was applied in the fixed monitoring scene. Therefore, we proposed a new stereo night dataset in this system, and as for our constant research on this project over the past year, we have expanded the dataset. This dataset was captured with a binocular network camera designed by us at 640×480 resolution and included some common outdoor surveillance scenarios, such as buildings entrance, main street, etc. To the best of our knowledge, this is the first night stereo video surveillance dataset to address and analyze the performance of state-of-the-art pedestrian detection algorithms. At present, we have posted this stereo night dataset on a website: https://xdfhy.github.io/. Furthermore, due to the constant research in this project, we will continue to expand this dataset.

### 3.2. System Performance Evaluation

**Figure 5:**
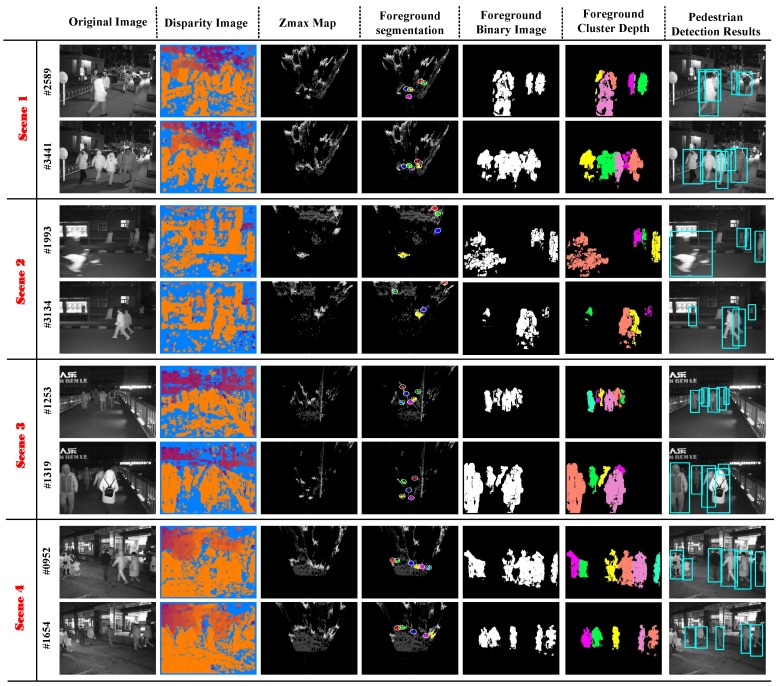
Pedestrian detection in four scenes at night, including campus entrances, main street, footpath and flyover. These scenarios cover the challenges of traditional methods of pedestrian detection, such as congestion, object occlusion and dim light.

In this work, the performance of our system was evaluated on four broadly-used scenes at night, including the entrance of campus, dim footpath, a crowded flyover, main street. These scenes covered the challenges of traditional pedestrian detection methods at night such as object occlusion and less texture information (as shown in Figure 5).

As shown in Figure 5, we can see that the detection results of our nighttime pedestrian detection system were effective in a complex environment. Figure 5 not only shows our experimental process on the four scenes, including the original image, disparity map, Zmax map and foreground segmentation on the Zmax map, but also shows the three resulting images containing the foreground binary image, foreground cluster depth and pedestrian detection results. Then, we see the original images in Figure 5 and specifically analyzed these four experimental scenes. First, we take a look at Scene 1: we know this experiment was carried out at a campus entrance, where surveillance systems were often established for student safety. There was a serious occlusion problem owing to the large number of people and cars, and we know the obtained original image had little texture information, which were challenges for the traditional nighttime pedestrian detection technologies. Scenes 2 and 3 are dim and narrow paths, and we can see that there is severe occlusion, as well as dim light, which brought difficulties to the depth calculation. Therefore, the disparity map had many invalid points. Scene 4 is a main street, surrounded by many shops. This scene had many chaotic lights, and the nighttime pedestrian detection system could be used to monitor the flow of shop staff, which is promising for the future.

In other words, these four scenes are representative night monitoring scenes, including some challenges usually encountered by traditional night pedestrian detection systems. To observe the output results of our system in Figure 5, we see that our nighttime pedestrian detection system can effectively detect the pedestrians in the scene when faced with these problems, the number of targets detected being quite high and the false detection rate low. We can analyze the performance of this algorithm more specifically according to Figure 5. On the one hand, our pedestrian detection system can easily solve the partial occlusion, such as Frame 2589 in Scene 1, Frame 3134 in Scene 2, and Frame 1319 in Scene 3. This is because our system used a binocular camera to obtain the depth information of foreground targets and segmented the foreground pedestrian in the 3D space. Furthermore, we know that although the two targets were overlapping in the original image, the two targets usually were not at the same depth, and the distance may not be small in the 3D space. Like Frame 2589 in Scene 1, from the foreground cluster depth, we can see that the depths of the two overlapping objects were not the same, and they were separated on the Zmax map. Notice that the target was undetectable when it was blocked too much, because the target’s valid 3D points were too few.

On the other hand, our system was not very sensitive to the intensity of light in the scene. From Figure 5, we can find that the pedestrian detection results are robust and effective in the environment with chaotic lights, such as Scene 4, and the dim environment such as Scenes 2 and 3. The system could even detect pedestrians that were hard to see by human eyes. That was because our method focused on pedestrian profile information, so it was not strict in the quality of the disparity map. For example, see Frame 1993 in Scene 2 and Frame 1253 in Scene 3: the color and texture features of the original image are not obvious, and there are many invalid points in the disparity map; however, the overall contours of the pedestrian on the disparity map are relatively complete, and the target points are clustered together and their number greater than on the Zmax map; Therefore, our pedestrian detection system could get a robust and acceptable pedestrian detection result in the dim night. What is more, our system was not so strict on the height of the camera so as to keep more information about the people. We can see the face information and people’s posture information in Figure 5. This information can be used for other studies on images. Generally speaking, all of the results of detection showed that our system could effectively solve the problems encountered by pedestrian detection algorithms at night, and the performance of our system was efficient and robust.

### 3.3. Comparing with State-Of-The-Art Methods

For further analysis, the proposed system was evaluated through a comparison with the three classic and state-of-the-art pedestrian detection methods. These three methods are mainstream solutions of pedestrian detection in surveillance scenes and include:

(1) Background subtraction methods: the widely-used background subtraction algorithms, such as ViBe [17] and fast MCD [18]. ViBe [17] has the advantages of a small amount of calculation, a small memory footprint, high processing speed and the detection of good features. Fast MCD [18] models the background through the dual-mode Single Gaussian Model (SGM) with age for foreground object detection on non-stationary cameras.
(2) RGB-D methods such as [30]: In [30], the algorithm uses a stereo vision system called subtraction stereo, which extracts a range images of foreground regions, then the extracted range image is segmented for each object by the clustering method.
(3) Learning-based detection methods such as the classic algorithm DPM [21] and the current popular deep learning algorithm YOLO2 [26]: DPM [21] is a successful target detection algorithm and has become an important part of many classifiers, segmentation, human gesture and behavior classification. YOLO2 [26] is a state-of-the-art, real-time object detection algorithm that can detect over 9000 object categories.

**Figure 6:**
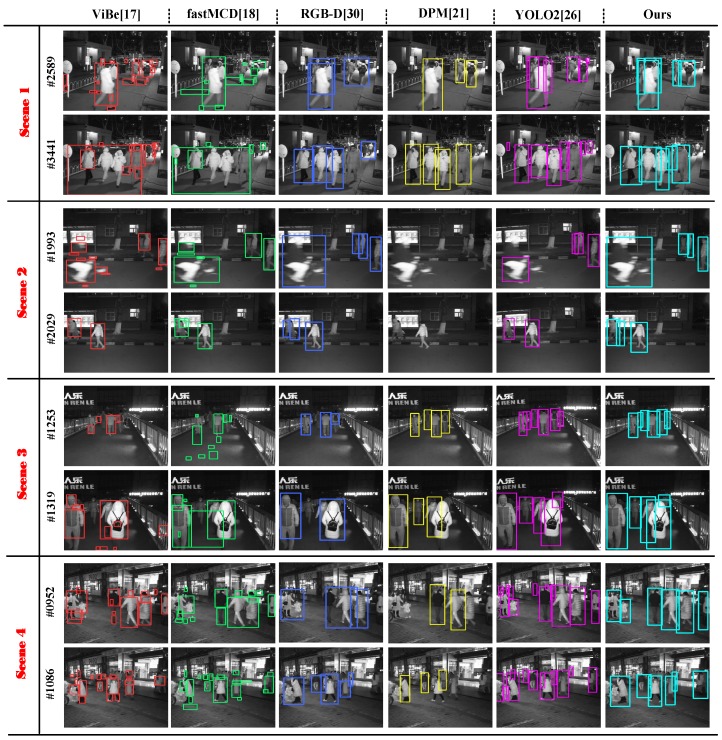
The detection results of four typical datasets showed the difference between our approach and the Visual Background extractor (ViBe) [17], the Deformable Parts Model (DPM) [21], fast MCD [18], You Only Look Once Version 2 (YOLO2) [26] and RGB-D [30]. The four scenarios include (**a**) school entrance; (**b**) footpath; (**c**) flyover and (**d**) busy street.

We chose the five algorithms mentioned above for comparative experiments and selected four sets of scenarios for testing to obtain the different detection results (as shown in Figure 6). As for the specific implementation of the experiment, first, the code implementation of ViBe [17] came from [37]. The parameters in the code are the standard parameters of the algorithm, such as the number of samples per pixel and the sub-sampling probability, etc. The fast MCD [18] code was from [38], then we followed the steps to implement the code and complete the test, and the parameters of the code did not change, which maintained the original settings. In addition, because these methods deal with a single image, we selected the left rectified image as the input of the algorithm. Second, the RGB-D method [30] is a stereo algorithm, and the input is left and right rectified images. What is more, this method’s source code cannot be found on the Internet, so we wrote the code and completed the test according to the algorithm flow described in [30]. Finally, the DPM [21] algorithm code was from [39]; this algorithm requires only a single image, and the code could be used directly to complete the experiment after the input was changed to our left rectified image. As for YOLO2 [26], the code was downloaded from the official website [40]. In general, a large amount of data needs to be trained to generate the weights when using this algorithm. However, there are many good training weights for pedestrian detection on this official website. In the experiment, we chose the current mainstream weights directly (YOLOv2 [26]) and the left rectified image as the input, and these weights have been tested and found to be very effective.

Various metrics can be used to assess the output of a pedestrian detection algorithm given a series of final detection images. These metrics usually involve the following quantities: the number of true positives (TP); the number of false positives (FP); and the number of false negatives (FN) [17]. Thereafter, we could calculate the precision, recall and F1-measure (precision and recall weighted average). For the purpose of comparison, we selected four scenes and 400 frames in each scene. Then, we obtained these parameter values from the detection results (as shown in Table 2).

As presented in Figure 6, first, we could know that ViBe [17] and fast MCD [18] easily split one target into multiple targets and regarded the several overlapping targets as one target. What is more, these two methods lost many blocked targets due to the poor image quality at night. Second, the detection results of DPM [21] were acceptable, but there were some false detections and some targets missed owing to serious occlusion. Third, as for the method in [30], we know moving foregrounds were segmented firstly in each individual camera by grey level background subtraction, and then, stereo matching was applied only on the segmented foreground image to obtain the depth of the human. Therefore, the gray level background subtraction easily failed due to poor color discrimination of infrared images at night, which leads to a severe decrease in the performance of the following stereo matching and pedestrian detection. Therefore, we found that there were some targets missed in Figure 6, such as Frame 3441 in Scene 1 and Frame 952 in Scene 4. Finally, we can see that the detection results of YOLO2 [26] were robust and effective. However, it still cannot overcome the inherent problem of a single camera without 3D information. Its performance will be reduced in a scene with severe occlusion, such as Frame 2029 in Scene 2. Furthermore, the infrared image had less texture information, blurred contours and poor color discrimination at night, resulting in some foreground objects being similar to the background, so there were some false detections and some targets missed, such as Frame 952 in Scene 4.

After that, we obtained the statistics of the detection results and performance metrics of various algorithms. From Table 2, we could ascertain that the total number of people in the experimental scene was 1476. Fist of all, it can be seen that the precision of our system was the highest in these methods up to 95.40%, and the precision of DPM [21] and RGB-D [30] was also good at about 90%. By contrast, the precision of YOLO2 [26] was not high, which was 74.47%, and this is because YOLO2 [26] had many erroneously-detected targets, although the FP of YOLO2 [26] occupied first place among these methods. Then, we found the precision of ViBe [17] and fast MCD [18] to be the lowest at less than 35%. Thereafter, looking at the calculated recall in the table, we know that the recall of YOLO2 [26] was the highest at up to 85.97%; the recall of our algorithm took second place, which is 71.61%; and the recall of ViBe [17], DPM [21] and fast MCD [18] was about 50%. We know the larger the value of precision and recall, the better the performance of the algorithm. Therefore, we chose the F1-measure of all of the algorithms synthetically, and we could know that our algorithm and YOLO2 [26] were the most effective at about 0.8; the RGB-D [30] method and DPM [21] were also acceptable; and ViBe [17] and fast MCD [18] were the lowest at less than 0.45.

It is noted that the difference in the F-measure between our approach and background subtraction such as ViBe [17] was obtained by comprehensive experiments in many nighttime scenes. Our method was essentially different from the traditional background subtraction method, which led to such a huge difference in performance.

Many of the traditional background subtraction methods such as ViBe [17] are color based. They learn a background model with input image sequences and segment the foreground through comparing the color difference of the input image and background image. Obviously, these color-based background methods are very sensitive to illumination changes and cluttered background. In addition, their performance will be significantly reduced in the case of a crowded scene with an occluded or even invisible background. Different from traditional methods, our algorithm was based on the three-dimensional spatial structure information of the scene. Once a moving person appeared, the height structure in the three-dimensional space would change simultaneously. Since our method only relied on the spatial information of the scene, it was not affected by light changes, and it was still very robust in the crowded scene because of the possession of spatial information.

Finally, we could ascertain the processing speed of every algorithm from Table 2, as well as the computational efficiency: the proposed system ran on a notebook with an Intel i7-6700 CPU and 8 GB RAM. Our system could process at 25.3 fps on this platform, so this method basically met the system’s real-time requirements. ViBe [17] is a fast method that can process 33.3 fps on the same platform, but the precision was too low to be used directly. The DPM [21] processed 1.7 fps on the same platform, which is too slow. The YOLO2 [26] is very slow on the CPU, so this method usually processes on the GPU, and this method processed at 25 fps on GPU Navida Geforce gtx1060 and 6GB video memory. In summary, we could know that our nighttime pedestrian system performed well and significantly outperformed some recently published state-of-the-art methods, including commonly-used background subtraction algorithms such as ViBe [17] and fast MCD [18], feature-based detection algorithms such as DPM [21] and RGB-D algorithms such as the method in [30]. In addition, the performance of our system was comparable to the most popular deep learning method, YOLO2 [26], and sometimes outperformed it.

**Figure 7:**
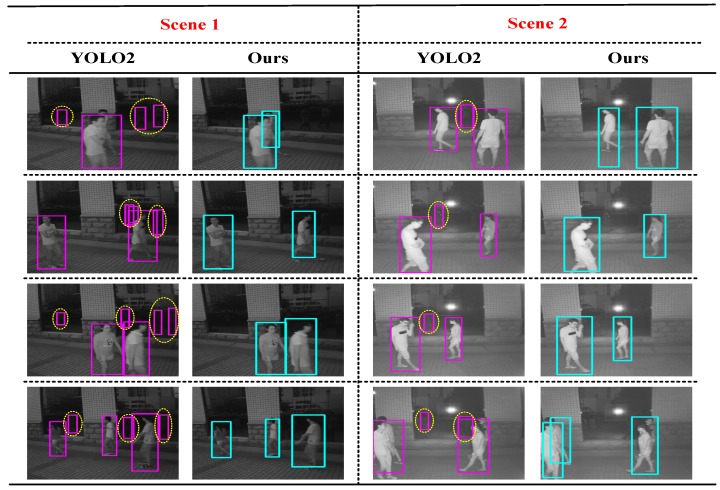
The detection results of YOLO2 [26] and our method in the nighttime scenes with some French windows. For pedestrian detection algorithms, this kind of scenario has a special challenge in addition to the common problems of the night scenarios, i.e., the presence of mirror images in the scene can interfere with the pedestrian detection.

In order to further compare with YOLO2 [26], we chose a special scenario to experiment with, and this scenario had severe specular reflection due to a mirror or window. As given in Figure 7, there are many large French windows outside the building, which are quite common in shopping malls and libraries, etc. We can see that pedestrians leave a clear mirror image on the window when they pass through this scene. From YOLO2 [26]’s detection results in Figure 7, we find out that YOLO2 [26] can easily detect these mirror images as pedestrians and once again found that this method cannot detect the blocked targets, which can lead to a significant reduction in the detection effect. Compared with YOLO2 [26], our method could acquire 3D information in the scene and set a monitoring range in 3D space to remove the French window when detecting pedestrians; thereby, we find the false detection rate to be low in Figure 7. What is more, we could see that this algorithm can solve partial occlusion problems and improve the detection rate. In other words, comparing all of the detection results of these two methods, we could recognize that our system works much better than YOLO2 [26] in this kind of scenario.

We summarize the characteristics of these two algorithms in Table 3. Firstly, the computing environment of these two methods was not the same, and YOLO2 [26] improved the speed with the help of the Graphics Processing Units (GPUs). In contrast, our system achieved real-time performance without GPU acceleration, so our binocular system would be more easily used in real life. Secondly, YOLO2 [26] is a feature-based detection method that only gets the bounding box of the object. Our approach was a pedestrian detection method based on the voxel surface model; in addition to getting the bounding box, we also obtained all of the moving foreground points’ information in the scene. Finally, our system used a binocular camera, so we could obtain the depth of all foreground points and the distance of the foreground targets. In other words, although their detection performance was quite good, our approach had lower requirements for the system and was more convenient. What is more, there was more information including the 3D information of the scene, foreground points and the distance of foreground targets, which was helpful for other studies in the scene. In addition to the detection results presented in the paper, we also tested several datasets and produced a video demo to demonstrate the effectiveness of our system at night. Please review it in the Supplementary Material.

## 4. Conclusions

In this paper, we proposed a novel near-infrared binocular camera for nighttime pedestrian detection, which was composed of two ordinary network infrared cameras. The camera was connected to the computer through a network cable, so it was easy to implement the outdoor nighttime pedestrian detection system through a small router. In this way, we could obtain the infrared image in the scene. Thereafter, we presented a background modeling method based on the voxel surface model for the nighttime pedestrian detection system, as well as a new updating strategy and a segment strategy using the extracted shadows. On the one hand, the method was carried out in 3D space, so our system more easily handled occlusion while minimizing the loss of additional pedestrian information. On the other hand, this method was not strict about the quality of the disparity map, and we could obtain an accurate result of pedestrian detection as long as the outline of pedestrians could be acquired, so our system had high accuracy for some environments at night, despite a low resolution image, a poor sense of hierarchy, less texture information, contour blur and severe occlusion.

In the experiments, we first evaluated the performance of our pedestrian detection system by carrying out testing on the datasets of various situations at night, and the experimental results demonstrated that the proposed system was efficient and robust. Thereafter, the effectiveness and efficiency of our system were validated in comparison with some recently-published state-of-the-art methods. The comparative experiments showed that our system outperformed common state-of-the-art approaches such as the background subtraction method, the feature-based method and the RGB-D method. What is more, the performance of our system was comparable to the performance of the deep learning method and sometimes outperformed it. Furthermore, our algorithm was not as computationally demanding as deep learning, which requires a GPU development environment and could get more information, which means that our system would be more easily used in real life for pedestrian detection and other foreground objects’ detection. Notice that this system was only applicable to a static platform for monitoring areas. If we were going to migrate the system to a mobile platform, such as installing a camera in a car for dynamic pedestrian detection, it would be necessary to compensate for the background model, which will be a new approach and may be attempted in the future. In future work, we will work on building a stereo video dataset in multiple indoor and outdoor surveillance scenarios. Moreover, because our pedestrian detection system could keep the 3D information of the pedestrian in the scene, we will attempt to perform people tracking and re-identification on the basis of the proposed work. Our system designed a novel pedestrian detection algorithm based on the voxel surface model for these application scenarios.

## Figures and Tables

**Table 1:** The binocular camera parameters.

Binocular Camera	Specification	Parameter
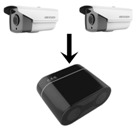	sensors	1/3${}^{\prime \prime }$ Progressive Scan CMOS
	day and night conversion mode	Infrared Cutfilter Removal(ICR) infrared filter
	infrared irradiation distance	25 m
	baseline distance	190 mm
	maximum resolution	2048×1536 at 25 fps
	working voltage	DC 12 V ± 10%
	maximum power consumption	7.5 W
	operating temperature	$-30{\;}^{\circ }$C∼60 ${}^{\circ }$C

**Table 2:** False alarm and missed rate of detection.

Algorithm	Total Targets	TP	FP	FN	Precision	Recall	F1-Measure	Speed (fps)
**ViBe [17]**	1476	714	2839	762	20.10%	48.37%	0.28	**33.3**
**DPM [21]**	1476	694	**35**	782	95.20%	47.02%	0.63	1.7
**fast MCD [18]**	1476	804	1677	672	32.41%	54.48%	0.41	18.2
**YOLO2 [26]**	1476	**1269**	435	**207**	74.47%	**85.97%**	0.80	25 (GPU)
**RGB-D [30]**	1476	953	126	523	88.32%	64.56%	0.75	20.0
**Ours**	1476	1057	51	419	**95.40%**	71.61%	**0.82**	25.3

**Table 3:** Algorithm performance comparison with YOLO2 [26].

Algorithm	Computing Platform	Speed	Sample Training	Foreground Binary Image	Foreground Depth Information	Bounding Box
**YOLO2**	Navida Geforce gtx1060	25 fps	*√*	×	×	*√*
**Ours**	Normal CPU	25.3 fps	×	*√*	*√*	*√*

## Supplementary Materials

The stereo night dataset are available online at https://xdfhy.github.io/.
